# The acute effects of multi-ingredient pre-workout ingestion on strength performance, lower body power, and anaerobic capacity

**DOI:** 10.1186/s12970-016-0122-2

**Published:** 2016-03-08

**Authors:** Andrew R. Jagim, Margaret T. Jones, Glenn A. Wright, Carly St. Antoine, Attila Kovacs, Jonathan M. Oliver

**Affiliations:** Exercise & Sport Science Department, University of Wisconsin – La Crosse, La Crosse, WI USA; Division of Health and Human Performance, George Mason University, Fairfax, VA USA; Kinesiology Department, Texas Christian University, Fort Worth, TX USA

**Keywords:** Ergogenic aid, Fatigue, Strength, Anaerobic performance

## Abstract

**Background:**

Multi-ingredient pre-workout supplements (MIPS) are popular among resistance trained individuals. Previous research has indicated that acute MIPS ingestion may increase muscular endurance when using a hypertrophy-based protocol but less is known in regard to their effects on strength performance and high intensity running capacity. Therefore, the purpose was to determine if short-term, MIPS ingestion influences strength performance and anaerobic running capacity.

**Methods:**

In a double-blind, randomized, placebo controlled, crossover design; 12 males (19 ± 1 yrs.; 180 ± 12 cm; 89.3 ± 11 kg; 13.6 ± 4.9 %BF) had their body composition assessed followed by 5-repetition maximum (5RM) determination of back squat (BS; 119.3 ± 17.7 kg) and bench press (BP; 92.1 ± 17.8 kg) exercises. On two separate occasions subjects ingested a MIPS or a placebo (P) 30-minutes prior to performing a counter movement vertical jump test, 5 sets of 5 repetitions at 85 % of 5RM of BS and BP, followed by a single set to failure, and an anaerobic capacity sprint test to assess peak and mean power. Subjective markers of energy levels and fatigue were also assessed. Subjects returned one week later for a second testing session using counter treatment.

**Results:**

MIPS resulted in a greater number of repetitions performed in the final set to failure in the BP (MIPS, 9.8 ± 1.7 repetitions; P, 9.1 ± 2; p = 0.03, *d* = 0.38), which led to a greater total volume load (set x repetitions x load) in the MIPS (753 ± 211 kg) compared to P (710 ± 226 kg; *p* =0.03, *d* = .20). MIPS ingestion improved subjective markers of fatigue (*p* = 0.01, *d* = 3.78) and alertness (p = 0.048, *d* = 2.72) following a bout of resistance training. An increase in mean power was observed in the MIPS condition (p = 0.03, d = 0.25) during the anaerobic sprint test.

**Conclusion:**

Results suggest that acute ingestion of a MIPS study may increase upper body muscular endurance. In addition, acute MIPS ingestion improved mean power output during an anaerobic capacity sprint test. However, the practical significance of these performance related outcomes may be minimal due to the small effect sizes observed. MIPS ingestion does appear to positively influence subjective markers of fatigue and alertness during high-intensity exercise.

## Background

A class of dietary supplements, which contains a variety of ingredients blended together, has become a popular ergogenic aid used by both elite and recreational athletes [1]. These supplements, referred to as multi-ingredient performance supplements (MIPS), are consumed under the assumption that they will improve subsequent performance post-ingestion [2]. Specifically, they are purported to improve strength, power, and work capacity during training sessions thereby potentially augmenting training adaptations over time. As a result, the majority of these products are often marketed toward resistance trained individuals. Common ingredients of MIPS include caffeine, branch chain amino acids (BCAA’s), creatine, and beta-alanine. Previous research has shown that a number of the individual ingredients contained in MIPS may improve performance as each ingredient is associated with a different physiological mechanism [3–6]. As an example, acute use of caffeine, which has been reported to improve muscular strength, power and endurance during high-intensity exercise, acts as a central nervous system stimulant, and may also enhance calcium release within skeletal muscle [7, 8]. The regular use of beta-alanine has been reported to improve the buffering capacity of skeletal muscle and enhance power output during high-intensity exercise [9, 10]. As a result this has led some to believe a synergistic effect may be present when specific ingredients are combined into a MIPS product. As evidenced, several studies have found similar improvements in acute exercise performance following ingestion of different MIPS supplements. For example, Hoffman et al. [11] noted greater lower body training volume when subjects were instructed to complete 6 sets of 10 repetitions for the back squat (BS) using a load corresponding to 75 % 1-repetition maximum (1RM). Similarly, Spradley et al. [12] reported a greater number of repetitions to failure performed during a set of leg press using 75 % 1RM. Further, Spradley et al. [12] noted an improvement in choice reaction speed and subjective markers of fatigue post- ingestion of a MIPS. These improvements in acute exercise performance may lead to enhanced training adaptations over time as previous research has shown that MIPS can increase muscular strength [13–16], fat-free mass [13, 14, 16–19] and anaerobic power [17] when combined with a structured training program.

The MIPS supplement from the current study is novel in that the label provides specific amounts of the ingredients as opposed to being classified as a proprietary blend, a common practice for supplement manufacturers. As dietary supplements are introduced to the market it is important to investigate their efficacy. At this time, we are not aware of any studies that have investigated the combination of ingredients in this MIPS supplement in relation to its potential impact upon strength performance, particularly when using a protocol designed to improve maximal strength rather than hypertrophy. Therefore, the purpose of the current study was to investigate the acute effects of a novel MIPS on strength performance, lower body power, and anaerobic capacity. In addition, we also sought to examine the effects of MIPS ingestion on ratings of mental alertness, energy, and focus in recreationally trained weight trainers. It was hypothesized that the acute MIPS would positively impact indices of alertness, fatigue, muscular power, and anaerobic capacity.

## Methods

### Experimental design

**Fig. 1:**
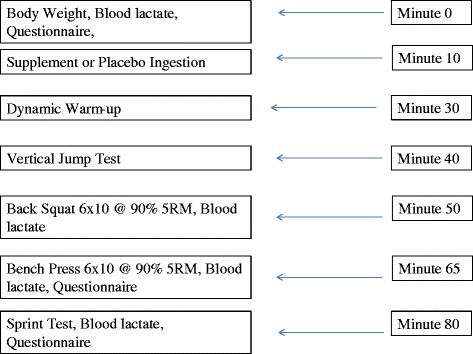
Timeline of experimental testing procedures

This study utilized a randomized, double-blind, placebo controlled cross-over design consisting of four separate testing sessions. Subjects completed a familiarization session consisting of a countermovement vertical jump test (CMVJ), sprint test against resistance on a non-motorized treadmill, and familiarization sets with the bench press (BP) and BS exercises in order to demonstrate proficiency with equipment and protocols. Subjects returned to the Human Performance Laboratory for baseline testing within one week of the initial meeting. At this time, height, weight, body composition, vertical jump height, and muscular strength were assessed in addition to a second familiarization trial on the sprint test. On a non-consecutive day (within 3–7 days), subjects returned to the laboratory to complete the first of two experimental testing sessions. Upon arrival to the laboratory for each experimental session, subjects were first weighed, had baseline blood lactate levels assessed and completed a questionnaire to assess subjective feelings of focus, energy, and fatigue prior to ingestion of the supplement or placebo. Subjects then consumed either 1 serving of the MIPS or a P. Twenty minutes following ingestion of the drink, subjects began a standardized warm up consisting of dynamic body-weight movements. Following the warm-up, subjects completed a CMVJ test, 5 sets of 5 repetitions for the BS and BP, and sprint test against resistance. Subjects returned to the laboratory a week later to repeat the experimental testing and ingested the opposite treatment. Fig. 1 outlines a summary of the timeline for the experimental testing sessions.

### Subjects

Twelve resistance trained, college-aged men who were Division III football players in their off-season (18.8 ± 1.2 yrs.; 180 ± 12 cm; 89.3 ± 11 kg; 13.6 ± 4.9 %BF) participated in this study. The players were classified as “grey shirts” as they were not officially on the team and were training for the upcoming season’s try out. Interested subjects first attended an informational meeting in which the details of participation were explained and written informed consent was obtained. All protocols were reviewed and approved by the Institutional Review Board at the University of Wisconsin – La Crosse and all subjects were 18 years of age or older. Entrance criteria included regular participation in a resistance training program utilizing the BS and BP exercises (>2 years). At the time of participation, subjects had been resistance training 2–3 times per week targeting upper and lower body muscle groups. Subjects were excluded if they had consumed any nutritional supplements containing caffeine, creatine or beta-alanine within the last 3 months. Health related concerns including metabolic disorders, auto-immune disease and/or clinically diagnosed hypertension were also grounds for exclusion. Furthermore, the MIPS supplement contained soy and was processed in a facility that processes peanuts, eggs, wheat and milk; therefore, anyone with allergies to these food items was prevented from participation in the study. Subjects were instructed to maintain regular eating and exercise habits throughout the duration of the study with the exception of performing the exercises used in the study 48 hours prior to testing. Subjects were instructed to consume the same food prior to each testing session and arrive to the laboratory fasted for >3 hrs.

### Baseline testing procedures

#### Body composition

Height and body mass were determined to the nearest 0.1 cm and 0.1 kg, respectively; using a stadiometer (*Seca; Chino, CA*) and Seca physicians scale (*Seca; Chino, CA*). Body composition was then assessed via under water weighing (*Hydrostatic Tank, L.H. Wolfia, IN, USA*). Residual lung volume was first determined using an oxygen dilution technique using previously described methods [20]. Subjects were seated on a chair attached to a load cell scale for a minimum of six trials. An average of the three heaviest weights was recorded for a true estimate of underwater weight, which was then used for body density calculation. Body fat percent was then determined from body density obtained during underwater weighing and the Siri prediction equation [21].

#### Counter-movement vertical jump

Following body composition assessment, subjects completed a standardized 10 minute warm up consisting of dynamic body-weight movements. Subjects then completed a CMVJ test on a jump mat (*Just Jump System, Probotics, AL, USA*), which consisted of three attempts with the highest CMVJ being recorded for analysis and later converted to power (W) using previously described methods [22]. During the CMVJ, the counter movement was done to the subject’s self-selected depth and arm swing was allowed. If the third jump was higher than the first two, additional attempts were allowed with a maximum of 5 jumps permitted and 2 minutes of rest provided in between attempts.

#### Maximal strength testing

Five minutes following CMVJ assessment, subjects completed a 5-repetition maximum (5RM) test for the BS and BP exercise. The same researcher determined the progression strategy for the 5RM based on the subjects’ self-reported maximal value and corresponding load percentages. First, subjects completed two warm up sets of 5–10 repetitions at 40-60 % of their estimated 5RM separated by two minutes rest. Next subjects completed one to two sets of 5 repetitions at a load corresponding to 60-80 % 5RM with three minutes of rest in between. Subjects then began performing sets of 5 repetitions of increasing weight for determination of 5RM. Three to five minutes rest were provided between each successive set. All 5RM determinations were made within 3–5 attempts. To be considered a successful attempt, subjects were required to squat down until the tops of the thighs were parallel to the floor. The same laboratory personal provided a verbal “up” command during all testing of the BS exercise. The same protocol was used for BP 5RM determination and to be considered a successful attempt, subjects were required to lower the bar to their chest. All strength testing was completed on a Smith machine (*Plyometric Power System; Norsearch, Australia*) and supervised by the same researcher.

### Experimental testing sessions

#### Supplementation

The MIPS tested in this study was a commercially available product which was independently obtained. The ingredients are listed in Table 1._._ The placebo (P) was matched for flavor and color, containing 5 calories of maltodextrin (*Crystal Light©, Kraft Foods Group, INC, Northfield, IL, USA*). Prior to testing, the assigned supplement was prepared by an outside member of the research staff and delivered to the laboratory with the subject’s name on a shaker bottle in order to maintain a double blind procedure. Each powder was mixed in a shaker bottle with 16 oz. of cold water and ingested in a hallway outside of the laboratory within 5 minutes of initiating ingestion.

**Table 1:** Supplement Ingredients

Serving Size: 1 Scoop (26.5 g)		
Amount Per Serving	Amount	% Daily Value
Calories	15	†
Total Carbohydrates	4 g	1 %*
Sugars	2 g	†
Sodium (as sodium chloride)	130 mg	5 %
Creatine Hydrochloride	2 g	†
CarnoSyn® Beta-Alanine	2 g	†
Betaine (Trimethylglycine)	1.5 g	†
Taurine	1 g	†
N-Acetyl L-Cysteine	600 mg	†
AlphaSize® Alpha-Glyceryl Phosphoryl Choline	300 mg	†
Citrulline Malate	6 g	†
Beta vulgaris L. (beet root extract)	500 mg	†
L-Leucine	3 g	†
L-Isoleucine	1.5 g	†
L-Valine	1.5 g	†
L-Tyrosine	1.5 g	†
Caffeine Anhydrous	300 mg	†
Huperzine A	50 mcg	†
Bioperine® (Piper Nigrum Fruit Extract)	5 mg	†

#### Testing procedures

Upon arriving to the laboratory, subjects first completed the 4-item questionnaire. Subjects then ingested the supplement or placebo drink, assigned in a randomized fashion. Following a twenty minute passive rest period, subjects completed the same warm up and CMVJ test used during baseline testing.

Next, subjects completed 5 sets of 5 repetitions of the BS at 85 % of their baseline 5RM with 2 minutes rest between sets. In addition, they completed a 6^th^ set receiving instructions to complete as may repetitions as possible until fatigue while attempting to achieve maximal velocity during each repetition. Fatigue was defined as failure to achieve 70 % of their average power output on two attempts during the 6^th^ set. Following a 10 minute rest period, subjects completed the same protocol with BP. During all repetitions of the BS and BP protocol, bar velocity was assessed using a linear position transducer (*Tendo Fitrodyne, Tendo Sport Machines, Slovak Republic*) attached to the right side of the bar in accordance with manufacturer instruction. The reliability of the Fitrodyne has been previously reported and yielded an intraclass correlation coefficient (ICC) of R = 0.97 (95 % CI, 0.95–0.98) in resistance trained males for lower body exercises [23]. Total repetitions and training volume load (sets x repetitions x load) were recorded and used for later analyses.

Ten minutes following the last set of BP, subjects completed a 25-second maximal effort sprint test on a non-motorized treadmill *(Woodway Inc., USA)* against a workload set at 18 % of their body weight as previously described [24]. Subjects were given a 3-second countdown and instructed to sprint as fast as possible for the entire 25-seconds. All sprints were started from a self-selected crouched, split stance position. Dependent variables for the sprint test included peak power, mean power and total work completed over the 25 second sprint. This protocol has been shown to have high test-to-test reliability using a similar population for peak and mean power (*r* = 0.96-0.97; CV = 6-7 %) [24].

### Measurements

#### Blood lactate

Blood lactate was assessed at baseline, immediately following the last repetition of the 6^th^ set for the BS and BP exercises and immediately following the anaerobic sprint test. Samples were collected using Mumford Uni-stick 3 (*Owen Mumford, UK*) normal single use safety lancets and assessed using a handheld lactate analyzer (*Lactate Plus, Nova Biomedical, MA, USA).*

#### Questionnaires

To assess subjective feelings of energy, fatigue, alertness and focus for tasking, subjects completed a 4-item questionnaire (Table 2) consisting of a 5-point rating scale by verbal selection according to previously used methods [2]. Specifically subjects were asked to rate their response using verbal cues on a scale of 1–5 which corresponded to: 1 = very low; 2 = low; 3 = average; 4 = high; 5 = very high. Questionnaires were completed prior to ingestion of the drink, following the last set of bench press and immediately following the anaerobic sprint. The questionnaires were administered by the same researcher each time.

**Table 2:** Survey Questionnaire

Questions	Responses				
*Please rate your energy level*	Very Low	Low	Average	High	Very High
	(1)	(2)	(3)	(4)	(5)
*Please rate your fatigue level*	Very Low	Low	Average	High	Very High
	(1)	(2)	(3)	(4)	(5)
*Please rate your feelings of alertness*	Very Low	Low	Average	High	Very High
	(1)	(2)	(3)	(4)	(5)
*Please rate your feelings of focus for task*	Very Low	Low	Average	High	Very High
	(1)	(2)	(3)	(4)	(5)

### Statistics

Descriptive statistics (mean ± SD) were computed for all measures of physical characteristics and performance tests. Changes in velocity, questionnaire responses, and blood lactate concentrations during the BP and BS exercises were assessed by a two-way (time by treatment) repeated measures analysis of variance (ANOVA). In the case of a significant interaction a LSD post-hoc analysis was completed to determine where significance occurred. Comparisons of number of repetitions to failure, total volume load, lower body power and anaerobic sprint performance were assessed using a paired samples *t*-test. Effect sizes were calculated and a modified classification system (trivial, 0.0-0.2; small, 0.2-0.6; moderate, 0.6-1.2; large, 1.2-2.0; very large, >2.0; extremely large, >4.0 was used [25]. Alpha was set at *p* ≤ 0.05 for statistical significance. All analyses were conducted using the Statistical Package for the Social Sciences (*SPSS, Version 21.0; SPSS Inc., Chicago, IL*).

## Results

**Fig. 2:**
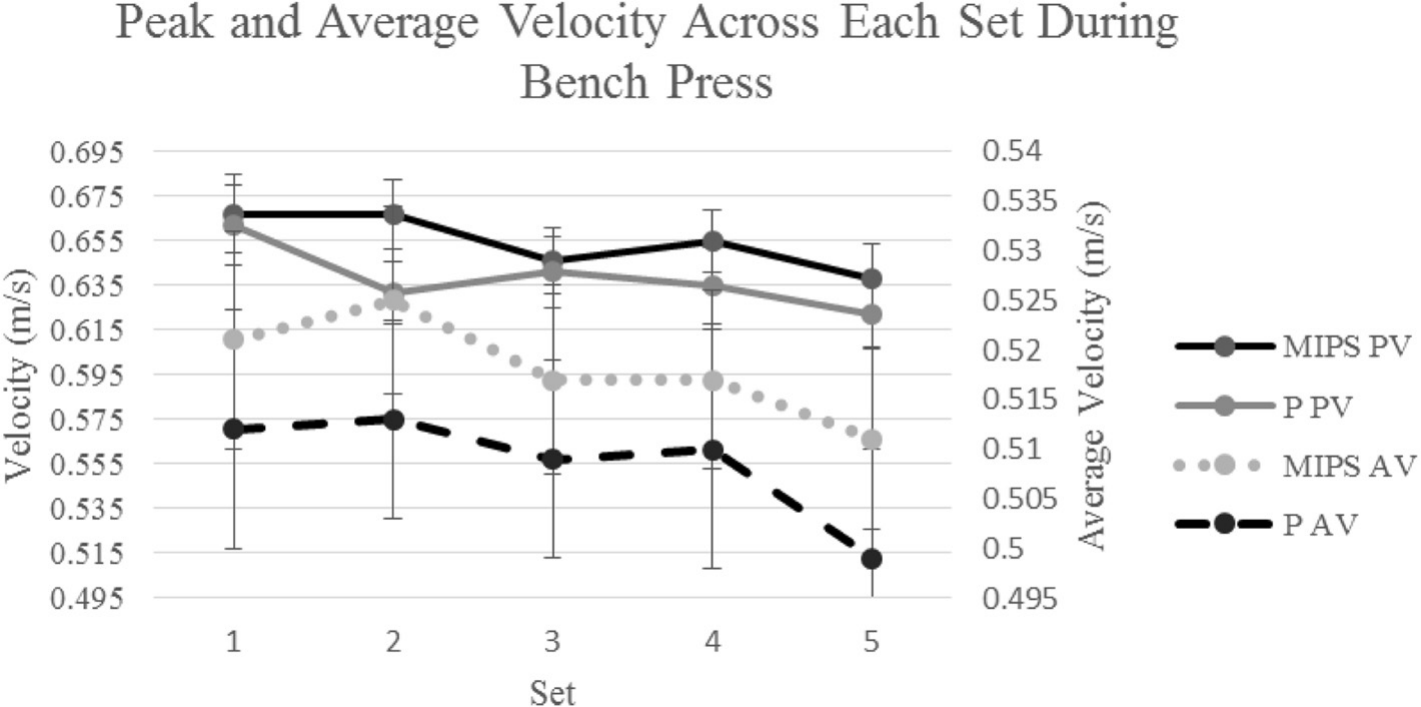
Peak and average Velocity Across Each Set During Bench Press. MIPS: Multi-ingredient pre-workout supplement; P: Placebo; PV: Peak velocity; AV: Average velocity

No adverse events or side-effects were reported following ingestion of the supplement or placebo. Mean 5RM BS and BP determined during baseline testing were 119.3 ± 17.7 and 92.1 ± 17.8 kg, respectively. Peak and average velocity declined (*p* < 0.001) when repetitions were collapsed and expressed as an average for each set by condition from Set 1 to Set 5 as presented in Fig. 2, with no differences observed between treatments. This was evidenced by a lack of a condition by set interaction for peak (*p* = 0.44) and average (*p* = 0.96) velocity, respectively. Further, when repetitions were collapsed and expressed as an average for each repetition, peak and average velocity in the BP also declined in an almost linear fashion reaching a point from repetition 3 to repetition 5 that was significantly lower (p < 0.001) compared to repetition 1 for both conditions.

**Fig. 3:**
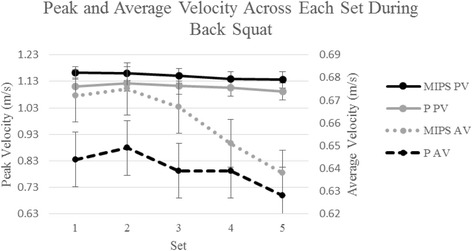
Peak and average Velocity Across Each Set During Bench Squat. MIPS: Multi-ingredient pre-workout supplement; P: Placebo; PV: Peak velocity; AV: Average velocity

When compared across BS sets, average velocity declined during subsequent sets for both conditions (*p* < 0.001) beginning at set 4 with no differences observed between conditions as evidenced by the lack of a condition by set interaction for peak (p = 0.85) or average velocity (*p* = 0.29) as depicted in Fig. 3. There was no significant main effect for treatment during the BS (MIPS 1.15 ± 0.052 vs P 1.109 ± 0.062 m/s; *p* = 0.073, *d* = 0.72) although a small effect size was observed. There was a significant main effect for time when examining peak velocity changes during the BS (*p* < 0.001). When collapsed across all sets and expressed as an average for each repetition during the BS, peak and average velocity were again significantly lower (*p* < 0.001) beginning at repetition 3 to repetition 5.

**Fig. 4:**
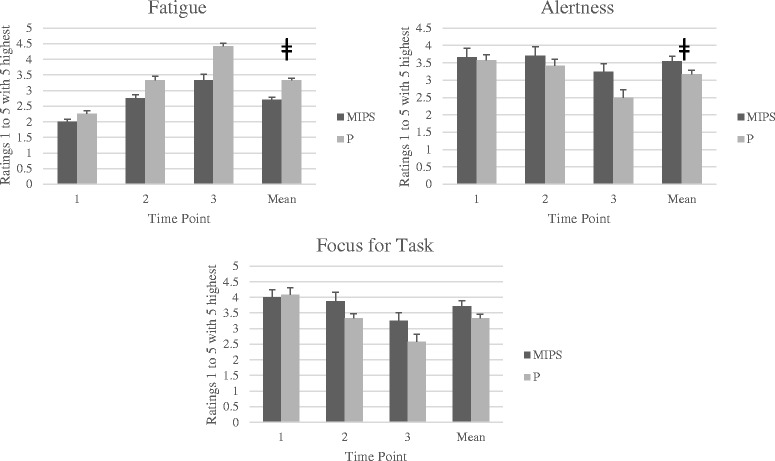
Questionnaire Responses. MIPS: Multi-ingredient pre-workout supplement; P: Placebo; PV: Peak velocity; AV: Average velocity.ǂ = Significant difference between MIPS and P

Following MIPS ingestion, subjects reported less fatigue compared to placebo as evidenced by a significant main effect for condition (MIPS 2.70 ± 0.19 vs. P 3.33 ± 0.14; *p* = 0.01, *d* = 3.78). Similar findings were reported in regard to feelings of alertness with the MIPS exhibiting higher ratings of alertness compared to P condition (MIPS 3.54 ± 0.15 vs. P 3.17 ± 0.12; *p* = 0.048, *d* = 2.72). A condition x time effect was observed for feelings of focus for task and Post hoc analysis revealed the MIPS condition exhibited greater feelings of focus for task after the sprint testing (MIPS 3.25 ± 0.25 vs P 2.58 ± 0.23; *p* = 0.013). No significant differences between condition were observed regarding feelings of focus for task (MIPS 3.71 ± 0.18 vs. P 3.33 ± 0.14; *p* = 0.072). A main effect for time was observed for energy levels (*p* = 0.01), fatigue levels (*p* < 0.001), feelings of alertness (*p* = 0.02) and feelings of focus for task (*p* = 0.004). Fig. 4 presents a summary of the questionnaire responses for fatigue, alertness and focus for task comparing differences between treatment conditions and time.

Blood lactate levels increased at each time point (*p* < 0.001); however, no time x condition effects were observed for changes in blood lactate (p = 0.54). Mean values for BS and BP repetitions to failure, CMVJ, and power are included in Table 3. MIPS resulted in a significantly greater number of repetitions to failure performed in the final set in the BP (MIPS, 9.8 ± 1.7 vs. P, 9.1 ± 2.0 repetitions; *p* = 0.027, *d* = 0.38) resulting in a greater total volume load (set x repetitions x load) in the MIPS (753 ± 211 kg) compared to P (710 ± 226 kg; *p* = 0.032, *d* = 0.20). No difference was observed in BS repetitions to failure (MIPS, 11.8 ± 3.9 vs. P, 11.0 ± 3.6 repetitions; *p* = 0.180, *d* = .21) or total volume load (MIPS, 1159 ± 359 vs. P, 1089 ± 364 kg; *p* = 0.186, *d* = 0.19).

**Table 3:** Summary of repetitions to failure and CMVJ performance

Variable	MIPS	PLA
BP to failure	9.8 ± 1.7	9.1 ± 2.0*
BS to failure	11.8 ± 3.9	11.0 ± 3.6
CMVJ (cm)	65.2 ± 7.0	65.8 ± 8.0
CMVJ Peak Power (W)	6470 ± 895	6513 ± 898
CMVJ Mean Power (W)	3415 ± 487	3438 ± 483

MIPS: Multi-ingredient pre-workout supplement; P: Placebo; PV: Peak velocity; AV: Average velocity; BP: Bench press; BS: Back squat; CMVJ: Counter-movement vertical jump

*Significant at p < 0.05

There were no significant differences in lower body peak (*p* = 0.584, *d* = 0.04) or mean power (*p* = 0.584, *d* = 0.04) as determined by CMVJ. A significant increase in mean power was observed in the MIPS condition (*p* = 0.034, *d* = 0.25) during the anaerobic sprint test. No significant differences were observed for any of the remaining anaerobic sprint performance variables. A summary of the anaerobic sprint performance variables is presented in Table 4.

**Table 4:** Performance variables for sprint test

Variable	MIPS	PLA
Total Work (W)	107.1 ± 4.8	106.7 ± 5.3
Peak Power (W)	1934 ± 379	1918 ± 376
Mean Power (W)	1468 ± 304	1397 ± 257*
Fatigue Index	0.448 ± 0.1	0.548 ± 0.2
Peak Velocity (m/s)	5.1 ± 0.2	5.1 ± 0.2
Mean Velocity (m/s)	4.4 ± 0.2	4.3 ± 0.2
Peak Force (W)	516 ± 122	533 ± 160
Mean Force (W)	332 ± 59	322 ± 52

MIPS: Multi-ingredient pre-workout supplement; P: Placebo

*Significant at p < 0.05

## Discussion

The primary purpose of the current study was to investigate the acute effects of an MIPS on strength performance, lower body power, and anaerobic capacity as well as its effects on ratings of mental alertness, energy, and focus in recreational weight trainers. To the best of our knowledge this is the first study to examine the effects of MIPS ingestion on strength performance using a common protocol designed to elicit strength adaptations (i.e., 5 x 5 @ 85 % 5RM) [26]. In addition, we wanted to utilize a protocol that would elicit a high degree of fatigue yet still allow for completion of the protocol that was determined through prior pilot testing. Previous research has indicated that declines in velocity during resistance training can be an indicator of fatigue [27] and in the current study a near linear decrease in peak velocity from repetition 1 to repetition 5 was observed in both the BS and BP, suggesting the current protocol was successful in eliciting fatigue. Even though the effect of MIPS ingestion on BS performance was not statistically significant, there was a small effect size observed (*d* = 0.72) for a condition effect, resulting in an improved ability to maintain peak velocity during back squatting. As a result, it appears that MIPS ingestion may attenuate the fatigue that is typically observed when performing several repetitions in succession however the practical implication of this outcome may be minimal due to the small effect size. These findings are in accordance with Gonzalez et al. [28] who reported significantly greater increases peak and mean power during 4 sets of 10 repetitions using 80 % 1RM at for the BS or BP exercise following ingestion of MIPS. The ability to maintain a higher working velocity and power output during multiple sets throughout a strength training session could potentially result in enhanced training adaptations in strength and power over time [29] however the direct effects of long-term MIPS ingestion warrants further investigation.

Additionally, based upon the results of the current study it appears as though acute ingestion of a MIPS may improve upper body muscular endurance and ultimately training-volume load as the MIPS condition yielded a greater number of repetitions in the final set to failure for the BP. However, again as a result of the small effect size observed (*d* = 0.38), the practical significance of this finding may be minimal. A positive effect was not observed with the BS exercise following MIPS ingestion, which is in opposition to previous findings. For example, Spradley et al. [12] found a significant improvement in the number of repetitions completed to failure for the leg press, at 75 % 1RM (SUP: 13 ± 6; PL: 11 ± 3), but not for BP following ingestion of a MIPS. It is important to note that Spradley et al. [12] used a lighter load and assessed lower body performance using the leg press rather than the back squat which could potentially explain the opposing findings in the current study as previous research has suggested that differences in repetitions to failure may arise when different modes of exercise are selected [30]. Further, Gonzalez et al. [28] observed a significant improvement in the number of repetitions when subjects were instructed to attempt 4 x 10 of either BP or BS (self-selected by subjects) at 80 % of a pre-determined 1RM 10 minutes following ingestion of a MIPS in resistance-trained men. Hoffman et al. [11] also found a significant increase in the total number of repetitions completed at 75 % 1RM for the BS exercise 30 minutes following ingestion of a MIPS in resistance trained males. Again the lack of a significant improvement in lower body muscular endurance may be attributable to differences in protocols used, specifically as the current study incorporated a set to failure following 5 sets of 5 repetitions. Additionally, in the previously mentioned studies velocity of each repetition was not measured; however, the decline in velocity in the current study suggests subjects fatigued in a similar fashion with no significant difference between treatments. It is worth noting that previous research has found that different loading schemes elicit varying degrees of metabolic and neuromuscular fatigue [27] which may dictate whether or not MIPS ingestion may be effective in serving as an ergogenic aid for different training protocols however this is beyond the scope of the current study. Further, although the subjects in this study were collegiate athletes, the training status of the individuals likely did not influence the findings as previous research has shown that training status has minimal impact on the number of repetitions performed at a given intensity [31].

Previous reports [2, 12, 32] investigating the effects of MIPS ingestion on anaerobic capacity have yielded mixed results. The results of the current study suggest that MIPS may improve mean power during maximal effort sprint tests with no influence on peak velocity. However, with the small effect size observed (*d* = 0.25) the practical significance may be minimal and, therefore is supported by the equivocal results observed in other studies. For example, Spradley et al. [12] did not detect a significant difference in intermittent critical velocity and intermittent anaerobic running capacity during repeat high-intensity treadmill tests following ingestion of a MIPS. Similarly, Hoffman et al. [2] also found no significant improvement in anaerobic power during a Wingate anaerobic power test in resistance trained males following ingestion of a MIPS.

The results of the current study also provide evidence that acute MIPS ingestion may positively influence measures of fatigue, feelings of alertness, and feelings of focus for task, which was demonstrated by the large effect sizes observed. Our findings are similar to those reported by Hoffman et al. [2], who observed a significant improvement in energy levels and focus for task following ingestion of a MIPS during repeated bouts of high intensity exercise. Spradley et al. [12] also found a significant improvement in subjective feelings of energy and fatigue following a muscular endurance test. In contrast, Gonzalez et al. [28] failed to observe an improvement in subjective feelings of energy, focus or fatigue following ingestion of MIPS using a similar protocol and questionnaire to that of the current study.

With the exception of caffeine, no other ingredient within the current MIPS has been shown to have an acute impact on performance in the absence of chronic loading. Therefore, it is likely that the primary active ingredient in the MIPS used is caffeine with a dose of 300 mg per serving, which equated to approximately 3.41 ± 0.45 mg/kg of bodyweight for the subjects in the current study. Other MIPS products contain varying amounts of caffeine, which may explain the conflicting findings. Several of the additional active ingredients of MIPS products (e.g., creatine, beta-alanine) have also been shown to improve anaerobic capacity when consumed for a longer duration [10, 33] through enhanced buffering capacity and intramuscular phosphate content and, therefore, perhaps a longer supplementation period would have resulted in an augmented anaerobic sprint performance.

Due to the lack of information in regards to the combination of the individual ingredients and their exact formulations used in other supplementation studies, it is difficult to speculate on the efficacy of individual ingredients or how one product compares to another as manufacturers commonly list their ingredients in “*proprietary blends*.” Further research, which selectively excludes certain ingredients from MIPS, is needed to specifically identify which ingredients serve as primary influential factors. Furthermore, differences in experimental design and/or training programs make it difficult to compare one product to another across multiple studies. However, based on the results of the present study it appears as though the combination of the ingredients in this commercially available MIPS may improve BS performance during subsequent sets in addition to improving mean power output during lower body anaerobic activities and positively influence subjective measures of fatigue and alertness during exercise.

## Conclusion

Based upon the results of the current study, ingesting a MIPS prior to a training session does not appear to attenuate fatigue-induced declines in velocity during a 5 x 5 lifting protocol for upper body exercises but may improve lower body performance. Further, MIPS ingestion likely has minimal influence on muscular endurance as a result of the low to moderate effect sizes observed for repetitions to failure and training load. Similarly, MIPS ingestion does not appear to substantially improve anaerobic capacity during sprint-based activities. MIPS ingestion does have a positive impact on subjective measures of fatigue and alertness, which could help provide motivation to individuals completing periods of intense training. Over time, these improvements could potentially augment training adaptations; however, additional research is needed to examine the long-term effectiveness of MIPS ingestion on performance and fatigue.
